# Breast Cancer and Nutrition: A Paradigm for Prevention in 3D Across the Life Course

**DOI:** 10.3389/fonc.2020.00129

**Published:** 2020-02-18

**Authors:** Michele R. Forman

**Affiliations:** Department of Nutrition Science, Purdue University Cancer Center, Center for Aging and the Life Course, Purdue University, West Lafayette, IN, United States

**Keywords:** nutrition, prevention, life course, paradigm, breast cancer

## Abstract

Breast cancer, the most common cancer in women worldwide, has recognized reproductive and anthropometric risk factors including age at menarche and adult height. Yet the age *when* a woman attains her adult height or experiences menarche for example is simply the timing of the major life event at the end of a long trail of exposures that began *in utero*. The objective of this article is to investigate through a review of the literature the role of nutrition in breast cancer prevention through three dimensions (D). Each D offers a different lens. The First D identifies *windows/ages* of exposures or conditions that convey vulnerability or protection from breast cancer. The Second *D* addresses the *intensity and duration* of the exposure; and the (*Third* D) examines the pace, i.e., how rapid or slow the young woman experiences her growth and development. Birthweight illustrative of the First D reveals a strong signal across the life course on BC risk, but the risk group varies from low to high birthweight. Stressful life events like being a pubertal aged girl living in a household with an unemployed father during the Great Depression or high levels of environmental contaminants exposure are representative of the Second D. Height velocity at specific ages and weight loss in postmenopausal years are illustrative of anthropometric trajectories that reveal an adaptive biosystem that provides a contextual state to interact with the other two Ds. This article presents a new paradigm of nutrition and breast cancer prevention through the lens of three very different dimensions. It is the premise of this article that all three dimensions are essential tasks to tease apart the life course and identify windows for preventive strategies.

Breast cancer is the most common cancer in women across the world (1). A family history of breast cancer (BC), high breast density, reproductive risk factors including early age at menarche, late age at menopause, older age at first birth, and nulliparity, as well as being tall, moderate to high alcohol consumption, being physically inactive and menopausal status specific-body mass index are a constellation of recognized risk factors influencing BC risk (2, 3). Yet the age *when* a woman attains her adult height or experiences menarche for example is simply the timing of the major life event at the end of a long trail of exposures that began *in utero*. The tempo of height velocity and the peak height velocity that end in a woman's adult height, and the age of first birth and pace of occurrence (i.e., time interval between first and last births) are essential components to understanding the cumulative risk from adult height and parity on BC risk (4). Indeed *profiling* a woman's linear growth trajectory *from birth across her life course* may likely be key to identifying and understanding strategies for BC prevention.

Hormonal exposures begin *in utero*. Proxy markers including the maternal pregnancy comorbidity of preeclampsia and an infant's birthweight are indicators of the hormonal milieu in fetal life. Estrogen, progesterone and insulin-like growth factor 1 (IGF-1) levels in cord blood vary by birthweight and preeclampsia exposure; they may set the baseline concentrations of hormones for breast cancer (5, 6). Each hormone has proliferative effects on the breast and concentrations vary dramatically by race-ethnicity, phase of the menstrual cycle, and parity (7–9). Haiman's ethnic- specific investigation of hormones by phase of the menstrual cycle in ovulatory Latina, non-Hispanic whites (NHW) and non-Hispanic Black (NHB) women revealed higher follicular and luteal phase estradiol concentrations in NHB women than Latinas and NHW; and in turn, Latinas had higher levels than NHW (10). In the multi-ethnic cohort of postmenopausal women, Japanese American and NHB women had higher estrogen levels than NHW (11). The absolute concentration of and timing of a hormone trajectory may be due to genetic and environmental influences as illustrated by ethnic-group specific differences above that have implications for BC risk. Understanding hormone trajectories and the timing of changes in the trajectory by life stage may help in capturing the *cumulative load of hormonal insults* related to the incidence of premenopausal BC.

To achieve the goal of breast cancer prevention, we need to examine the arsenal of exposures (both preventive and adverse), the window of the life course for the exposure (or its proxy indicator like hunger or an economic depression), and the trajectory of growth and the hormonal tone in a woman. Nutrition is fundamental to BC prevention because a woman's body mass and height for example are the result of diet, physical activity, metabolism, hormones, and reproductive life events that are underlying her body mass index, linear growth and attained adult height. The four indicators of nutritional status– anthropometric, biochemical, clinical and diet- are typically measured at one point in time in research rather than repeated measures that capture trajectories and change over the life course. It is the intent of this article to focus on life course approaches to research in nutrition and BC. The objective of this article is to investigate the role of nutrition in breast cancer prevention through three dimensions (D). Each D offers a different lens. The First D identifies *windows/ages* of exposures or conditions that convey vulnerability or protection from breast cancer. The Second *D* addresses the *intensity and duration* of the exposure; and the (*Third* D) examines the pace i.e., how rapid or slow the young woman experiences her growth and development. Growth occurs with damage to DNA repair and other components like radical oxygen species in carcinogenesis. Examination of the growth trajectory may provide context for biosystemic aging and interact with the influence of an exposure through prolonging or shortening it or modifying its intensity of effect as evidenced in the other 2Ds. Pregnancy has commonalities to carcinogenesis, because growth factors, hormones, and molecular pathways are up- and down-regulated with gestation but in a “controlled sense.” Pregnancy is a hyperinsulinemic state, with hormones at the highest concentrations experienced by a women in her life. Therefore, growth and pregnancy have always been risk factors but not placed into the context of their trajectory in a life course approach. Encapsulating a life course approach to breast cancer through nutrition can offer a unique lens into prevention and provide strategies for intervention and further research.

## The First D: Windows of Exposure Across the Life Course (Figure 1)

The hormonal milieu in pregnancy/*in utero* offers a window of exposure for breast cancer. Hormone levels in pregnancy vary by race-ethnicity, birthweight and parity. Concentrations of free estradiol and percent free estradiol are higher in the first than subsequent pregnancies (12). Non-hispanic Black women have higher testosterone levels in pregnancy than Non-Hispanic whites or Asians (9). Estriol and sex-hormone binding globulin protein levels increase with each standard unit (112 and 75 g increase) of birthweight (13). Furthermore, cord blood insulin like growth factor-1 levels are significantly higher amongst the high birthweight than normal or low birthweight newborns (5).

**Figure 1:**
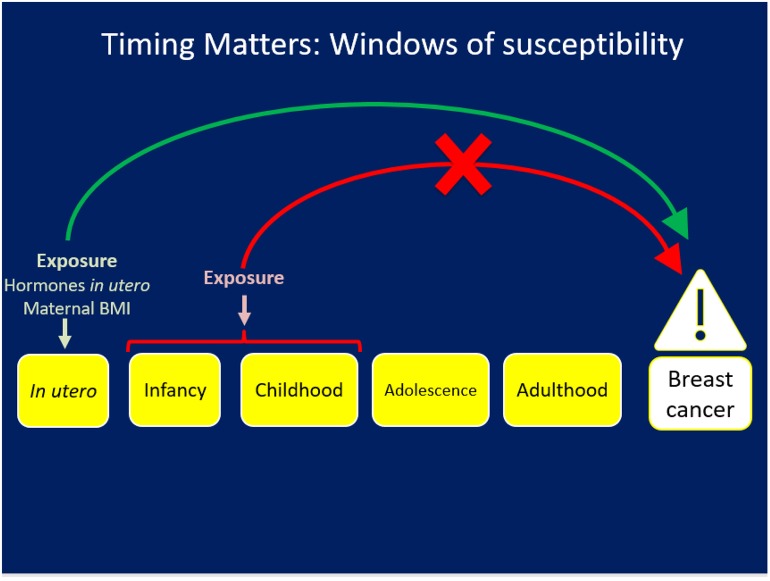
The First D: Exposures occurring during a specific window like *in utero* may have an impact on risk for chronic disease/breast cancer but the same exposure at a different life stage will not have the same impact.

Birthweight of the offspring is a proxy indicator for the fetal hormonal milieu and the nutritional status of the mother in pregnancy. Weighing 8.8 pounds or more at birth is associated with a 3.2-fold higher risk of early breast development (Tanner Stage 4–5) by 9–10 years among girls in the U.S. (14). Higher birthweight as illustrated by each 500 g increment is associated with a seven percent (95% CI; 1.02–1.13) risk for premenopausal breast cancer amongst Scandinavian women (15). A meta-analysis of birthweight and postmenopausal breast cancer revealed a 20% higher risk (95% CI 1.08–1.34) amongst those who weighed 4,000 grams or more at birth (16). Conversely low birthweight was associated with reduced risk (of a hazard ratio (HR) = 0.66; 95% CI: 0.47–93) of premenopausal breast cancer in the Nurses' Health Cohort Studies I and II (17). Birthweight reveals its signal through its effects on timing of breast development through to BC risk across the life course. In contrast, maternal pre-pregnancy body mass index and gestation weight gain were not associated with breast mammographic density in daughters of the index pregnancy in one study (18).

Evidence for *infancy* as a period of vulnerability for breast cancer arises in conjunction with *the third D* notably the trajectory of weight gain. Specifically, risk for breast development by 10.8 years in Norway varies by timing of peak weight gain in infancy and by maternal preeclampsia status. In a nested case-cohort study of preeclampsia, we report that peak weight gain during the third through 6th months of infancy in a daughter of a women with a normotensive pregnancy incurs a 1.87 risk for early breast development by 10.8 years. In contrast peak weight gain in the last 6 months of infancy in daughters of preeclamptic pregnancy has a 3.19-fold increased risk for early breast development (Thelus-Jean R 2009). Rapid weight gain in the first 4 months of infancy is associated with a 60% or higher risk for a diagnosis of benign breast disease (19). In contrast, other exposures during infancy such as infant feeding practices are not associated with risk for breast benign breast disease (20) or breast cancer (21).

Diet and body size in *childhood* are related to early breast development in Norway and percent breast density in the U.S. Specifically milk, butter and ice cream consumption at 3–5 years was inversely associated with early breast development in Norwegian girls aged 10.8 years (OR = 0.97, 95% CI: 0.95–1.00) after adjustment for birthweight, preeclampsia, weight, and height and other covariates (22). A recent systematic review concluded there was a likely association between childhood animal protein intake and *earlier puberty* assessed by age at menarche and age at peak height velocity (23). Finally the heaviest body size at age 10 as illustrated using the Stunkard images vs. the leanest body sized girls had a 5.9 fold (95% CI: −9.2–2.3) lower percent breast density when they reached ages 40–64 years, with 7.69 cm^2^ (95% CI: −13.9–0.63) smaller dense breast area, and 26.17 cm^2^ (95% CI: 9.42–43.58) larger non-dense area (24).

**Figure 2:**
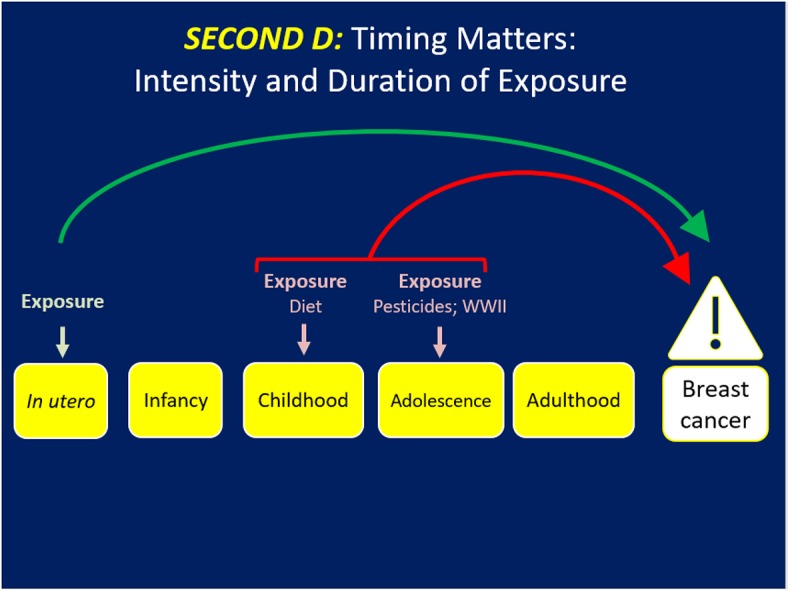
Timing matters but the intensity or concentration and duration of the exposure may dramatically influence risk of BC.

*The Second D (**Figure 2**) addresses the intensity and duration of the exposure* and offers a different lens into breast cancer prevention. Cohn et al. reported that women who were exposed to the middle and highest tertiles of DDT before 14 years of age had a 2.80 (95% 1.10–6.80) and 5.14 (95% CI 1.70–17.1) fold increased risk for breast cancer, respectively, compared to women in the lowest tertile of exposure at the same age. Those women exposed at or after 14 years had no risk of BC by tertile of exposure to DDT (25). Thus, early to late childhood when the breast is developing comprised the window of vulnerability for BC risk due to DDT exposure. Being in the middle and highest tertile of exposure to DDT during puberty was the marker for the intensity of exposure to confer BC risk.

Stressful life events in the family also offer a perspective on the timing of and intensity with which these events may have a role in breast cancer. For example, the Netherlands Cohort Study covered the era of the Great Depression 1929–32 through the hunger winter of 1944–45 that was rampant in certain regions of the Netherlands. In this cohort, if the father was unemployed during the Great Depression (1929–32) the daughter had a marginally reduced risk by 18% (95% CI 0.66–1.02) of breast cancer (26) Living in a city during World War II when a girl was experiencing a growth spurt was associated with a 28% (95% CI 0.54–0.97) lower risk of BC (26). Further living in a city during the hunger winter of 1944–45 was associated with a 51% (95% CI 1.06–2.17) higher risk of BC if the girl had completed her growth spurt. Therefore, the Netherlands cohort study reveals that the type, timing, and intensity of life stress events (the first and second D) can be associated with higher or lower risk of BC.

**Figure 3:**
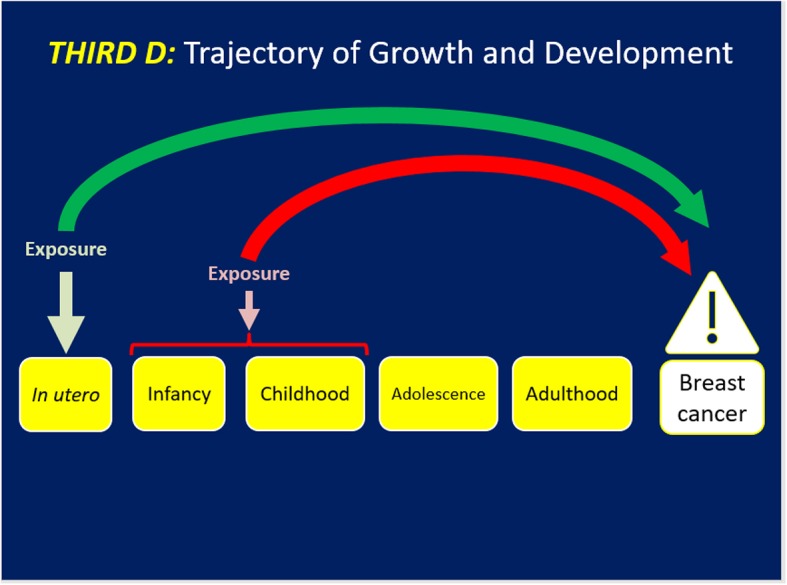
Trajectories of growth and development may reveal how the biosystem has adapted to cumulative hormones and growth factors that may influence BC risk. These trajectories may also set the stage for exposures identified in the first D and/or stressful life events in the second D to have an impact on BC.

*The third D (**Figure 3**) examines the effects of how rapidly or slowly a girl/woman experiences her linear growth and weight trajectory and/or hormonal and pubertal development and their implications for BC risk*. This D is revealed in a life stage-specific lens for BC risk with a strength that can be manifest across life stages (27–29). The first study appeared in the work by Ahlgren et al. amongst 117,415 Danish women with 3,340 BC cases that demonstrated the independent effects of a 10–17% range in higher BC risk for: the high birthweight, those with peak linear growth from 8 to 14 years i.e., puberty and attained adult height on BC risk (30). This landmark research introduced linear growth trajectory as a key component of BC risk. Berkey et al. investigated in the Growing Up Today Study (GUTS) that height at age 10 and peak height velocity were associated with risk for benign breast disease (31). Li et al. reported in the Vitamin and Lifestyle study that reaching the age of maximum height by 12 years conferred a 50% (95% CI 1.10–1.90) higher risk of BC than those who reached maximum height by age 17 years after adjustment for covariates (32). Rosner examined weight and weight changes in early adulthood and later BC risk using the NHSII (33). Weight at age 18 was inversely associated with pre and postmenopausal BC (HR per 30 Kg = 0.52, 95% CI: 0.39–0.71; HR = 0.82 95% CI: 0.72–0.92). In contrast, weight gain since age 18 was positively associated with ER+/PR+ postmenopausal BC (HR per 30 kg = 1.50 (95% CI: 1.36–1.65) but not with ER+/PR- or ER-/PR- BC. Overall 17% of ER+/PR+ BC was attributable to weight gain of >5 kg since age 18. In a multi-center analysis of pooled cohort studies, premenopausal BC risk was inversely associated with BMI at ages 18–24 years (HR per 5 kg/m^2^ difference 0.77 95% CI 0.73–0.80) (34). Associations were strongest for ER+/PR+ subtype of BC but the HR did not vary by other BC risk factors nor for BMI later in adulthood. Chlebowski et al. recently reported that among a cohort of 61,335 healthy postmenopausal women without breast cancer, those who experienced a weight loss of five percent or more over 3 years had a HR of 0.88 (95% CI: 0.78–0.98) for BC compared to those whose weight remained stable, revealing how weight loss in the postmenopausal years can prevent BC (35). Another recent work by Luo et al. demonstrated in the Women's Health Initiative (WHI) that being low birthweight conferred a lower risk of postmenopausal BC by 22% (95% CI: 0.79–0.99). The effect of birthweight on postmenopausal BC risk was appreciably mediated by adult height (40% proportion mediated) and weight at baseline ages of 50–79 years (21% proportion mediated). Obesity in late adulthood (>50 years) was associated with higher risk of BC. Furthermore, weight gain in adulthood over a 25 years period was also positively associated with BC risk regardless of the age/life stage (36).

## Summary and Conclusions

This paper presents a life course approach to nutrition and breast cancer in three dimensions. The evidence base for each D and the picture puzzle that appears by addressing all three Ds offers a unique lens into nutrition and BC. The first D focuses on windows of vulnerability for indicators of nutritional and hormonal status. Birthweight reveals a strong signal across the life course on BC risk, but the direction of the associations are not consistent. Specifically the signal for high birthweight on BC appeared in some (15, 16) but no other studies (17, 36) thereby casting a doubt whether high birthweight can be a proxy indicator for fetal hormonal milieu (5). Self-reported birth weight data in Xu et al. (16), Michels et al. (17) and Luo et al. (36) and enrollment of different birth cohorts influence the overall distribution of birthweight (and concomitant percent low or high birthweight) in each cohort study that may contribute to the inconsistency of the findings. The appreciable proportion of the birthweight effect on BC risk that is mediated my adult height and weight lends credence to the need for repeated measures of anthropometrics to recognize the trajectory and strength of the signal from birthweight across the life course (36).

Weight gain (and the pace of weight gain) during specific months in infancy influences breast development, and the risk for benign breast disease. The turning point for weight and its direct influence on BC risk arises from the data on the independent effect of weight at age 18 and of weight gain over the adult years on BC risk. Stunning evidence now appears that BC can be prevented by weight loss over a 25 years period capturing peri-and postmenopausal intervals; these data are primarily based on NHW in the U.S. and need further research in other race-ethnic groups and countries. How much weight is sufficient to prevent BC and how long the weight loss needs to be sustained to reduce risk are other elements that need flushing out.

Height in the absolute sense and in multiple manifestations of the linear growth trajectory has a strong signal for BC. Height velocity, age of peak height velocity, and attained height directly influence BC risk. Illuminating what these markers of BC risk mean is a challenge. The insulin-like growth factor 1 signaling pathway and genes are contributors to height but different ages have different patterns of linear growth. For example, infants typically gain weight before a linear growth spurt, however this pattern is not so evident in adolescence, when leptin and IGF-1 work in tandem during puberty. What are the underlying pathways at these stages lending themselves to different phenotypic hormonal precursors to linear growth? How do they relate to BC risk?

The timing and intensity of exposure to pesticides and stressful life events influence BC risk. DDT exposure at a certain level and before 14 years, i.e., puberty exhibited a signal for BC risk; any exposure at 14 years or later let alone exposure to a lower level had no effect. Likewise being in a household with an unemployed father during the Great Depression or experiencing hunger in an urban area during World War II was sufficient to be an indicator of risk for BC. It appears that three parameters–age, the intensity of the exposure and the timing during development– are key to identifying the components in the life course that are related to BC risk later in life.

This article presents a new paradigm of nutrition and breast cancer prevention through the lens of three very different dimensions. It is the premise of this article that all three dimensions are essential tasks to tease apart the life course and identify windows for preventive strategies. The picture puzzle has the potential for enrichment by examination of the gene-environment interactions in diverse populations and the examination of the epigenetic influences from diet, pesticides, and other environmental exposures.

## Data Availability Statement

The datasets generated for this study are available on request to the corresponding author.

## Ethics Statement

Ethical review and approval was not required for the study on human participants in accordance with the local legislation and institutional requirements. Written informed consent for participation was not required for this study in accordance with the national legislation and the institutional requirements.

## Author Contributions

The author confirms being the sole contributor of this work and has approved it for publication.

### Conflict of Interest

The author declares that the research was conducted in the absence of any commercial or financial relationships that could be construed as a potential conflict of interest.
